# Apremilast reduces co-occurring alcohol drinking and mechanical allodynia and regulates central amygdala GABAergic transmission

**DOI:** 10.1172/jci.insight.189732

**Published:** 2025-04-22

**Authors:** Valentina Vozella, Vittoria Borgonetti, Bryan Cruz, Celsey M. St. Onge, Ryan Bullard, Roman Vlkolinsky, Diego Gomez Ceballos, Angela R. Ozburn, Amanda J. Roberts, Roberto Ciccocioppo, Michal Bajo, Marisa Roberto

**Affiliations:** 1 Department of Translational Medicine, Scripps Research Institute, La Jolla, California, USA.; 2 Department of Behavioral Neuroscience at Oregon Health & Science University and VA Portland Health Care System, Portland, Oregon, USA.; 3 Animal Models Core Facility, Scripps Research, La Jolla, California, USA.; 4 Pharmacology Unit, School of Pharmacy, University of Camerino, Camerino, Italy.

**Keywords:** Neuroscience, Public Health, Addiction, Pain, Pharmacology

## Abstract

The FDA-approved phosphodiesterase type 4 (PDE4) inhibitor, apremilast, has been recently investigated as a pharmacotherapy for alcohol use disorder (AUD) with promising efficacy in rodent models and humans. However, apremilast’s effects on mechanical allodynia associated with AUD as well as distinct responses of this drug between males and females are understudied. The present study examined the behavioral and electrophysiological effects of apremilast in Marchigian Sardinian alcohol-preferring (msP) rats and their Wistar counterparts. We used a 2–bottle choice (2-BC) alcohol drinking procedure and tested mechanical sensitivity across our drinking regimen. Spontaneous inhibitory GABA-mediated postsynaptic currents from the central nucleus of the amygdala (CeA) following apremilast application were tested in a subset of rats using ex vivo electrophysiology. Transcript levels for *Pde4a* or -*4b* subtypes were assessed for their modulation by alcohol. Apremilast reduced alcohol drinking in both strains of rats. Apremilast reduced mechanical allodynia immediately after drinking, persisting into early and late abstinence. Apremilast increased GABAergic transmission in CeA slices of alcohol-exposed Wistars but not msP rats, suggesting neuroadaptations in msPs by excessive drinking and mechanical allodynia. *Pde4* subtype transcript levels were increased in CeA by alcohol. These results suggest that apremilast alleviates co-occurring excessive drinking and pain sensitivity, and they further confirm PDE4’s role in pain-associated AUD.

## Abstract 2

This report examined the behavioral and and electrophysiological effects of apremilast in a rodent model of high alcohol intake and enhanced pain sensitivity. Our results indicated that apremilast decreased alcohol intake and associated mechanical allodynia across groups of males and females in our genetically selected strain of rats. Apremilast increased GABAergic transmission in CeA slices of alcohol-exposed Wistars, but not msP rats, suggesting neuroadaptations in msPs by excessive drinking and mechanical allodynia. Pde4 subtypes transcript levels were increased in CeA by alcohol.

## Introduction

Alcohol use disorder (AUD) is a major global health and economic problem (1–3). Stress and anxiety are major factors that promote excessive alcohol consumption, as individuals may self-medicate to alleviate negative emotional states associated with these conditions (4, 5). As a result, AUD has a high comorbidity with depression, anxiety, and pain (6, 7). Furthermore, women and men experience AUD at different rates and present distinct sensitivity as well as putatively different physiological responses to anxiety and pain disorders (8–11). This poses etiological challenges for the treatment of AUD and necessitates the examination of distinct responsivity of pharmacotherapies between male and female patients. Furthermore, the neural mechanisms that mediate AUD and alcohol-related pain need additional examination to improve comprehensive clinical interventions.

Phosphodiesterase type 4 (PDE4) has recently been investigated as a target implicated in motivated behaviors for alcohol use (12–16). PDE4 is a class of enzymes responsible for degrading cyclic adenosine monophosphate (cAMP), a second messenger that controls the inflammatory cascade, and exists in 4 different subtypes (i.e., PDE4A/B/C/D) (17). PDE4 is predominately found in immune cells but is also abundantly expressed in the brain (15–17). A link between PDE4 and AUD was demonstrated in human genome-wide association studies (18, 19). Administration of apremilast, a nonselective PDE4 inhibitor that is FDA approved for the treatment of psoriasis and psoriatic arthritis, has shown efficacy in reducing alcohol intake in both human and rodent models (12, 20, 21). Apremilast decreases binge-like drinking and self-administration progressive ratio breakpoints in mice bred for alcohol intoxication (12). Apremilast also decreased stress- and dependence-induced alcohol escalation in mice (12). Lastly, apremilast reduced the action potential threshold of dopamine D1-type, but not D2-type, containing medium spiny neurons (MSNs) in the nucleus accumbens (NAc) (12). Because AUD is often comorbid with stress, anxiety, and/or pain, the role of PDE4 inhibition in brain regions more closely associated with these behaviors needs to be further explored.

The central nucleus of the amygdala (CeA) is a critical region involved in the behavioral output of alcohol addiction, stress, anxiety, and pain processing (22–24). The CeA also exerts sex-dependent modulation of alcohol drinking and pain (25–27). Specifically, the CeA modulates hyperalgesia, recruits pain-associated pathways, and promotes age- and sex-related differences in animal models of pain (27–30). Indeed, several preclinical studies have demonstrated that excessive alcohol intake predicts the development of mechanical allodynia (31–34). Mechanical allodynia is a hypersensitivity state that is defined as a painful sensation caused by innocuous stimuli (35). The neuronal networks of the CeA are mainly composed of inhibitory γ-aminobutyric acid (GABA) neurons (GABAergic neurons), and following stress and/or alcohol dependence, neuroadaptations occur via increases in GABAergic transmission (22, 23). However, the effect of PDE4 inhibition by apremilast in the CeA GABAergic system in models of co-occurring alcohol drinking and mechanical allodynia has not been studied yet.

The genetically selected Marchigian Sardinian alcohol-preferring (msP) rats are well characterized for their high alcohol intake, anxiety, and fear behavior (36, 37). Recently, we reported persistent pain in msP rats compared with their Wistar background counterparts, validating increased sensitivity to pain in this rat line (24). Thus, the present study examined the behavioral effects of apremilast on alcohol drinking and mechanical sensitivity (defined as allodynia) in msP rats versus their Wistar counterparts. We hypothesized that chronic alcohol would promote greater development in mechanical allodynia in msPs versus Wistars based on prior work, and that apremilast would block drinking and alcohol-related allodynia in a strain-dependent manner (24). Previous work showed that alcohol drinking alters brain *Pde4a* and -*4b* gene expression (12, 38, 39). Thus, we examined gene expression of *Pde4a* and -*4b* in the CeA and NAc and the acute effects of apremilast on CeA GABAergic synaptic transmission using electrophysiology, and we hypothesized that genetic and physiological disruptions of PDE4 would also occur in a strain-dependent manner (40, 41). Lastly, we included male and female groups across strains and drugs to understand sex differences produced by apremilast across our multidisciplinary study.

## Results

### Apremilast decreases voluntary 2–bottle choice (2-BC) alcohol drinking.

**Figure 1:**
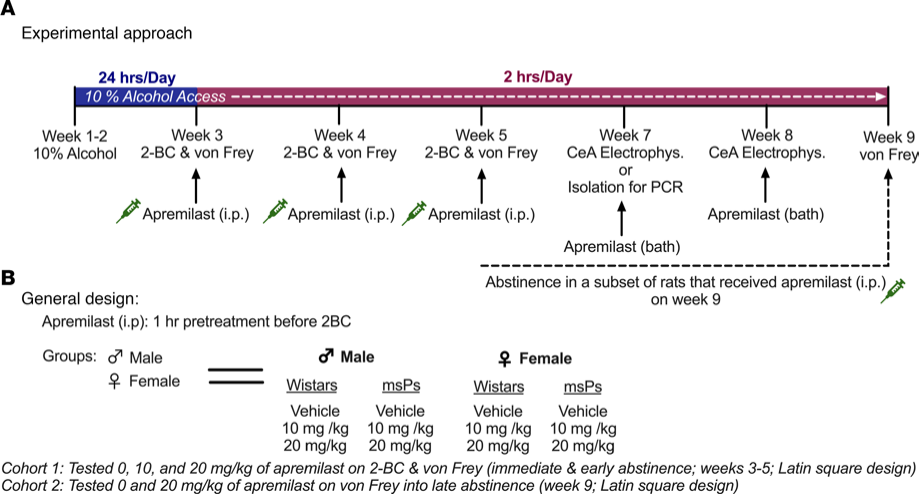
Timeline of apremilast testing on 2-BC alcohol drinking, mechanical allodynia, gene expression, and central amygdala (CeA) electrophysiology assessments. (**A** and **B**) Experimental approach (**A**) and design of apremilast dose regimen (**B**) on 2–bottle choice (BC) and von Frey testing across sex and strain. Rats first received 2 weeks of 10% alcohol for 24 hours/day. Starting on week 3, access to alcohol was reduced to 2 hours/day. During week 3–5, apremilast injections (0, 10, and 20 mg/kg; i.p.) was given in 3 cycles separated one week apart in a Latin square design. CeA electrophysiological recordings or isolation for PCR occurred on week 7, while recordings continued through week 8. A subset of rats were tested during late abstinence and received apremilast on week 9. All rats received apremilast injections 1 hour prior to testing on 2-BC, equivalent to 3 hours before von Frey testing on groups examined immediately after alcohol.

**Figure 2:**
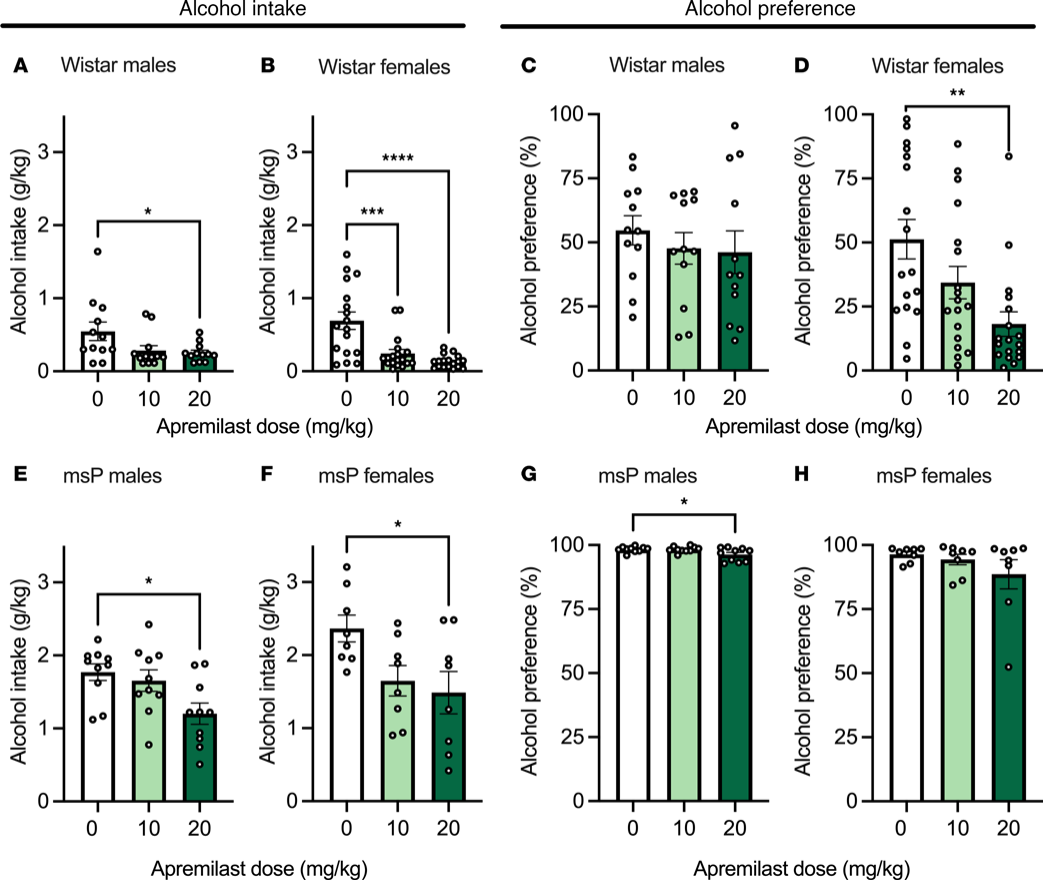
Apremilast decreases voluntary 2–bottle choice (2-BC) alcohol drinking across strain and sex. (**A**–**H**) Effects of apremilast (10 or 20 mg/kg) in the 2-BC drinking procedure (10% v/v alcohol) in Wistar (**A**–**D**) and msP (**E**–**H**) rats (*n* = 8–17 rats per group). (**A**) Wistar male alcohol intake, *F*_2,33_ = 3.61, *P* = 0.03. (**B**) Wistar female alcohol intake, *F*_2,50_ = 15.58, *P* < 0.0001. (**C**) Wistar male alcohol preference, *F*_2,33_ = 0.44, *P* = 0.64. (**D**) Wistar female alcohol preference: *F*_2,50_ = 6.74, *P* = 0.002. (**E**) msP male alcohol intake, *F*_2,27_ = 4.81, *P* = 0.02. (**F**) msP female alcohol intake, *F*_2,21_ = 4.05, *P* = 0.032. (**G**) msP male alcohol preference, *F*_2,27_ = 4.44, *P* = 0.020. (**H**) msP female alcohol preference, *F*_2,21_ = 1.29, *P* = 0.29. Results are expressed as mean ± SEM and analyzed as 1-way ANOVA followed by Dunnett’s multiple-comparison post hoc test. Significant difference relative to vehicle controls is denoted by *P < 0.05, **P < 0.01, *** *P* < 0.001, and ****P < 0.0001.

The regimen for apremilast administration on alcohol drinking and dose groups was included in our experimental timeline (Figure 1, A and B). Apremilast significantly reduced alcohol intake for males and females of both strains of rats (Figure 2, A, B, E, and F). However, apremilast only reduced alcohol preference among Wistar females (Figure 2D) and msP males (Figure 2G). Apremilast did not significantly alter alcohol preference for Wistar males (Figure 2C) or msP females (Figure 2H). No differences in water intake for males or females of either strain of rats were observed (Supplemental Figure 1, A–D; supplemental material available online with this article; https://doi.org/10.1172/jci.insight.189732DS1).

### Apremilast decreases mechanical allodynia across strain and sex immediately after alcohol exposure and into early abstinence.

Both male and female msP rats (Supplemental Figure 2, E and F) displayed increased mechanical sensitivity after alcohol consumption and during abstinence as compared with baseline, respectively. No differences were found between baseline across sexes for each strain (Supplemental Figure 2, A and D) or Wistar groups (Supplemental Figure 2, B and C).

**Figure 3:**
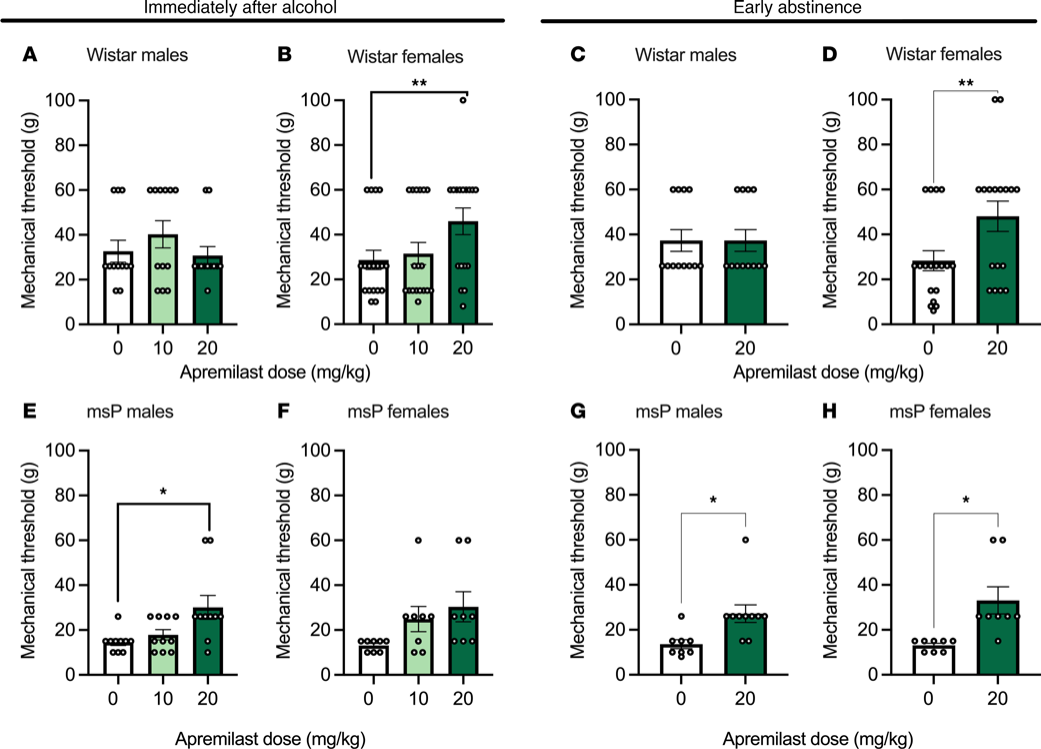
Apremilast decreases mechanical allodynia across strain and sex immediately after alcohol exposure and into early abstinence. (**A**–**H**) Effects of apremilast (10 or 20 mg/kg) on mechanical allodynia tested immediately after alcohol exposure (3 hours after injection, directly after 2-BC: left panel; 3–5 weeks into 2-BC) as well as during early abstinence (24 hours after injection: right panel; 5 weeks into 2-BC 24 hours after the last sessions) in Wistar (**A**–**D**) and msP (**E**–**H**) rats (*n* = 8–18 rats per group). (**A**) Wistar male mechanical threshold directly following 2-BC. Treatment *F*_1.918,21.10_ = 1.17, *P* = 0.32. Individual *F*_11,22_ = 1.58, *P* = 0.17. (**B**) Wistar female mechanical threshold directly following 2-BC. Treatment *F*_1.988,32.80_=7.99, *P* = 0.001. (**C**) Wistar male mechanical threshold in early abstinence; median (Mdn) = 0.0, Wilcoxon statistic (W) = 0.0, *P* > 0.000. (**D**) Wistar female mechanical threshold in early abstinence, Mdn = 16.0, W = 122.0, *P* = 0.002. (**E**) msP male mechanical threshold directly following 2-BC. Treatment *F*_1.27,11.40_ = 5.0, *P* = 0.04. (**F**) msP female mechanical threshold directly following 2-BC. Treatment *F*_1.70,11.93_ = 3.68, *P* = 0.06. (**G**) msP male mechanical threshold in early abstinence; Mdn = 11.0, W = 21.0, *P* = 0.03. (**H**) msP female mechanical threshold in early abstinence; Mdn = 16.0, W = 28.0, *P* = 0.01. Results are expressed as mean ± SEM and analyzed as a RM 1-way ANOVA or mixed-effects analysis as appropriate with Geisser-Greenhouse correction (**A**, **B**, **E**, **F**) followed by Dunnett’s multiple-comparison post hoc test and paired or unpaired *t* test (**C**, **D**, **G**, **H**). Significant difference relative to vehicle controls is denoted by *P < 0.05 and **P < 0.01.

To examine the effect of apremilast (0, 10, or 20 mg/kg; i.p.) on mechanical allodynia, von Frey testing was performed immediately after the 2-hour 2-BC drinking (3 hours after apremilast or vehicle injection) as well as in early abstinence (24 hours after injection). Immediately after alcohol drinking, apremilast (20 mg/kg) significantly reduced the alcohol-induced mechanical allodynia in female Wistars (Figure 3B) as well as male msPs (Figure 3E), but not in male Wistars or female msPs (Figure 3, A and F), when compared with vehicle-treated controls. This effect persisted in early abstinence 24 hours after alcohol removal (Figure 3, D, G, and H). There was no effect observed at the 10 mg/kg dose in Wistar or msP rats of either sex (Figure 3, A, B, E, and F). No effects were observed in Wistar males in early abstinence (Figure 3C).

### Apremilast reduces mechanical allodynia into protracted alcohol abstinence in female but not male msPs.

**Figure 4:**
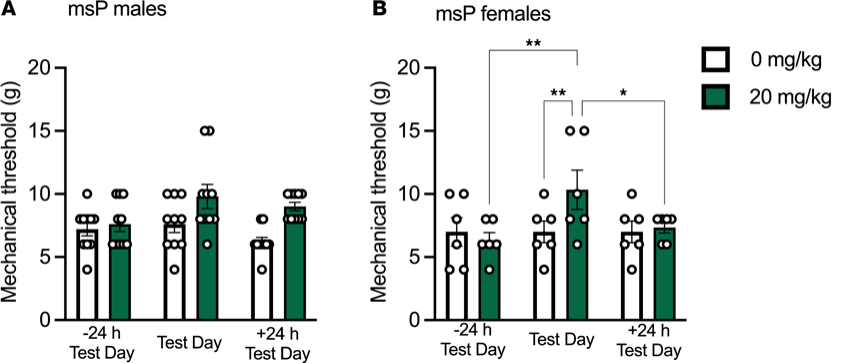
Apremilast reduces mechanical allodynia into protracted alcohol abstinence in female but not male msPs. Effect of apremilast (20 mg/kg) on mechanical allodynia in msP rats tested 4 weeks from the last 2-BC session (week 9; *n* = 6–10 rats per group). (**A**) msP male time course in mechanical threshold. Time, *F*_2,18_=3.64, *P* = 0.04. Treatment, *F*_1,9_=10.48, *P* = 0.01. Time × Treatment, *F*_2,18_=1.87, *P* = 0.183. (**B**) msP female time course in mechanical threshold. Time*, F*_2,10_=2.70, *P* = 0.11. Treatment, *F*_1,5_=0.84, *P* = 0.40. Time × Treatment, *F*_2,10_=4.87, *P* = 0.03. Results are expressed as mean ± SEM and analyzed as a RM 2-way ANOVA followed by Tukey’s multiple comparisons post hoc test. Significant difference is denoted by *P < 0.05 and **P < 0.01.

To evaluate apremilast’s effect on mechanical allodynia during protracted alcohol abstinence, apremilast (20 mg/kg) was administered 4 weeks after the last alcohol-drinking session only in msP rats (as they exhibited mechanical allodynia during abstinence; Supplemental Figure 2, E and F). Apremilast (20 mg/kg) reduced mechanical allodynia during protracted alcohol abstinence when compared with the vehicle-treated group in female but not male msP rats (Figure 4, A and B).

### Acute apremilast application increased spontaneous GABA transmission in ex vivo CeA slices of alcohol-exposed Wistar but not msP rats.

**Figure 5:**
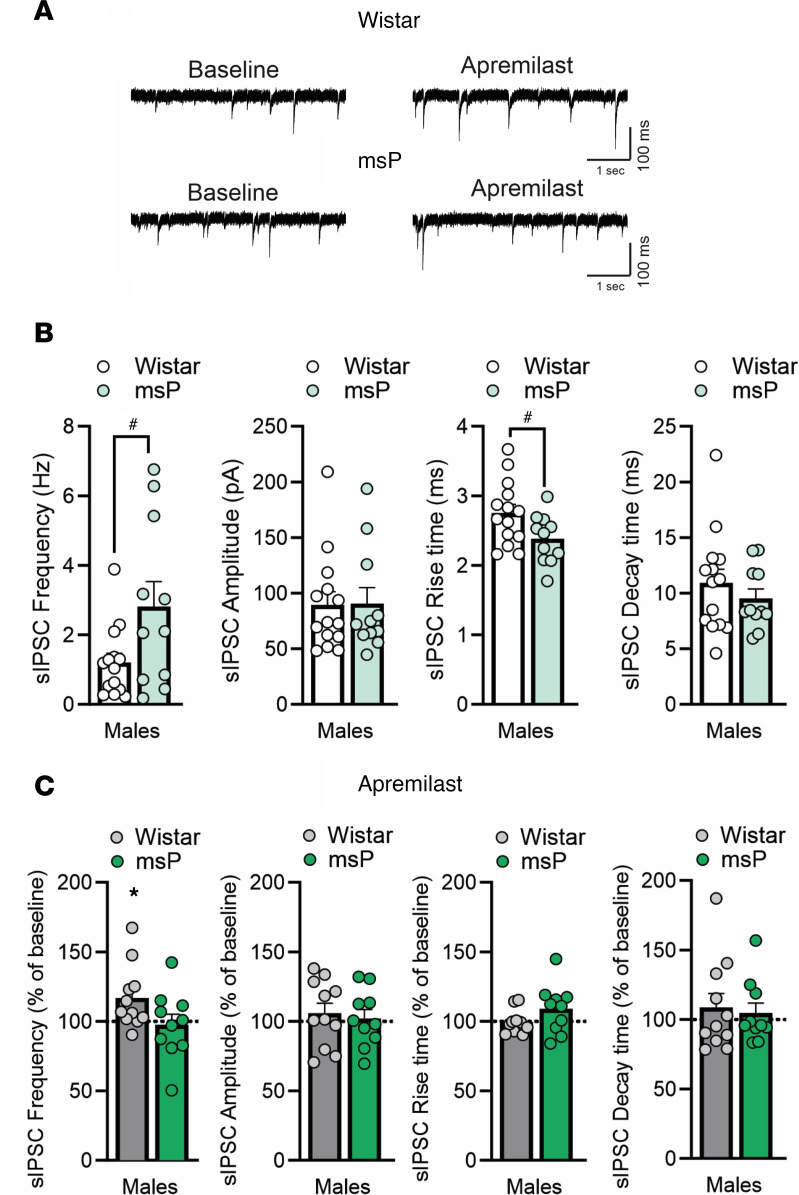
Acute apremilast application increased spontaneous GABA transmission in ex vivo CeA slices of male alcohol-exposed Wistar but not msP rats. (**A**) Representative baseline and during apremilast (1 μM) application of spontaneous inhibitory postsynaptic currents (sIPSC) in the CeA neurons from male Wistar and msP rats (*n* = 10–14 cells per group). All rats shared 2-BC alcohol access histories. (**B**) Baseline sIPSC frequency: *t*_23_ = 2.30, *P* = 0.03; amplitude: *t*_23_ = 0.07, *P* = 0.94; rise time: *t*_23_ = 2.21, *P* = 0.03; and decay time: *t*_23_ = 0.88, *P* = 0.38. (**C**) Effect of apremilast on sIPSC properties as compared with baseline frequencies: Wistar male, *t*_10_= 2.44, *P* = 0.03; all others not significant (ns) for amplitudes, rise time, and decay time. Results are expressed as mean ± SEM and analyzed as independent-sample *t* test for baselines or a 1-sample *t* test for apremilast effects. Significant difference is denoted by *P < 0.05 for apremilast effect, and ^#^*P* < 0.05 strain effect.

**Figure 6:**
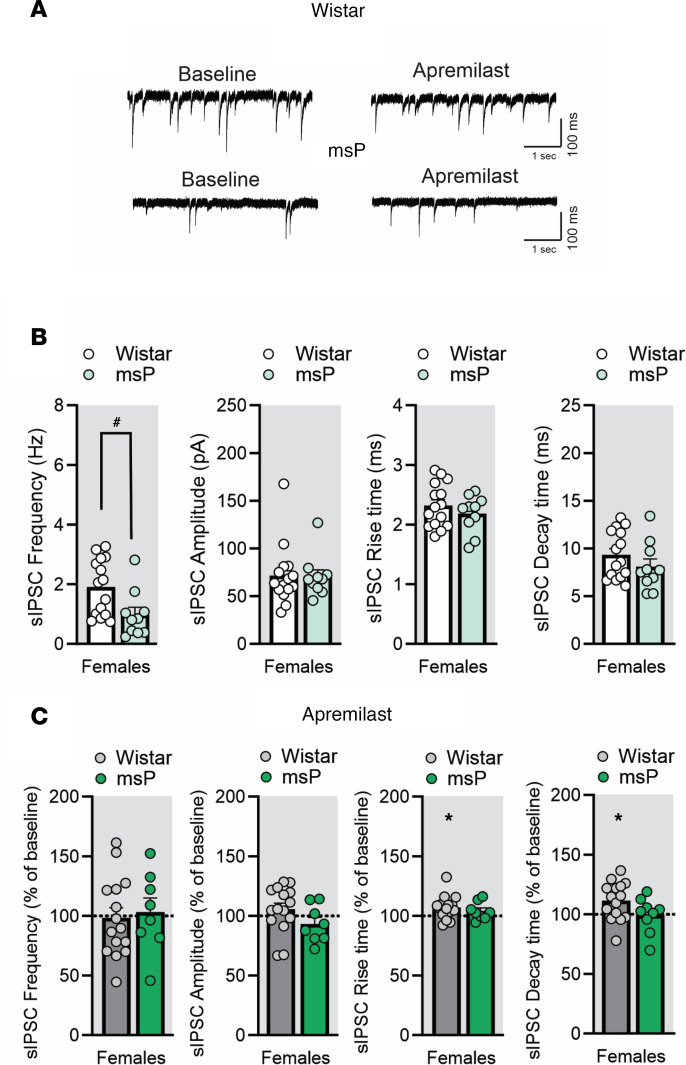
Acute apremilast application increased spontaneous GABA transmission in ex vivo CeA slices of female alcohol-exposed Wistar but not msP rats. (**A**) Representative baseline and during apremilast (1 μM) application of spontaneous inhibitory postsynaptic currents (sIPSC) in the CeA neurons from female Wistar and msP rats (*n* = 8–15 cells per group). All rats shared 2-BC alcohol access histories. (**B**) Baseline sIPSC frequency: *t*_23_ = 2.57, *P* = 0.01; amplitude: *t*_23_ = 0.08, *P* = 0.93; rise time: *t*_23_ = 0.96, *P* = 0.34; and decay time: *t*_23_ = 1.20, *P* = 0.23. (**C**) Effect of apremilast on sIPSC properties as compared with baseline rise time: Wistar female, t_14_= 2.75, *P* = 0.01, and decay time, Wistar female, t_14_= 2.78, *P* = 0.01; all others not significant (ns) for frequency and amplitude. Results are expressed as mean ± SEM and analyzed as independent-sample *t* test for baselines or a 1-sample *t* test for apremilast effects. Significant difference is denoted by *P < 0.05 for apremilast effect and ^#^*P* < 0.05 strain effect.

We used a random subset of rats for electrophysiological study to determine (a) potential baseline differences in CeA inhibitory signaling and (b) the synaptic effects of acute apremilast. We performed whole-cell patch clamp recordings of pharmacological-isolated GABA_A_-mediated spontaneous inhibitory postsynaptic currents (sIPSC) in the medial subdivision of the CeA from male and female Wistar and msP rats that underwent 2-BC. Notably, CeA neurons from msP males displayed significantly larger basal sIPSC frequency (2.82 ± 0.7 Hz) compared with 2-BC Wistar males (1.2 ± 0.3 Hz; Figure 5, A and B), suggesting increased GABA release in the msP strain (41). Furthermore, female msPs display significantly decreased GABA release compared with female Wistar (Figure 6, A and B). To determine the modulatory role of apremilast on inhibitory synaptic transmission, we applied 1 μM apremilast for 12–15 minutes on CeA sIPSCs. We found that apremilast significantly increased the sIPSC frequency (by 17% ± 6%) in the CeA of male Wistars (Figure 5C), and rise (by 7%; Figure 6C) and decay times (by 11%; Figure 6C) in female Wistars, without affecting any of the parameters in msP rats. These data suggest that apremilast significantly increases presynaptic GABA release in male and postsynaptic GABA_A_ function in female Wistars.

### Chronic alcohol drinking increases CeA Pde4a and -4b transcript levels in a sex and strain dependent manner.

**Figure 7:**
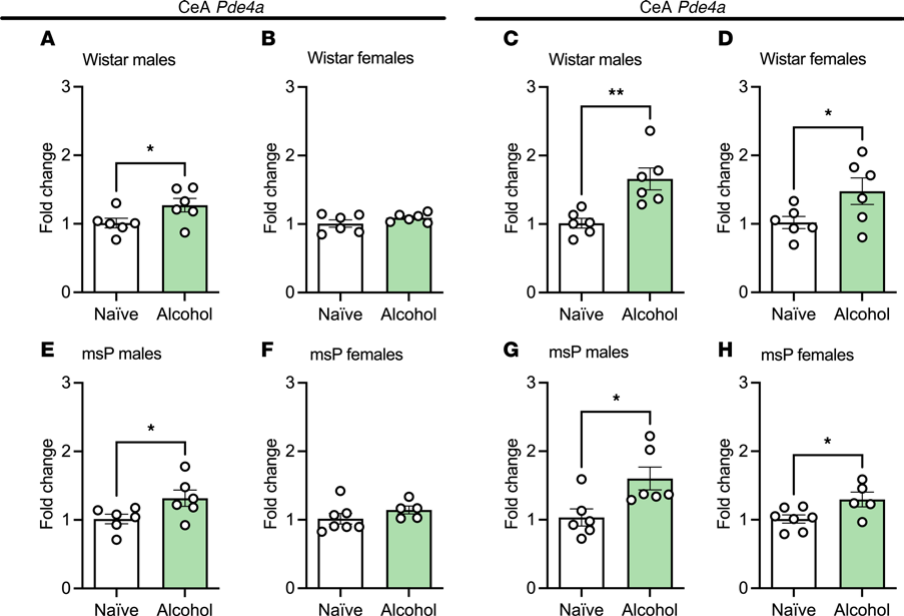
CeA *Pde4a* and *-4b* transcript levels are increased by chronic alcohol exposure across strain and sex. (**A**–**H**) Effects of chronic alcohol exposure (7 weeks) on CeA *Pde4a* (left panels) and *Pde4b* (right panels) transcript levels in Wistar (**A**–**D**) and msP rats (**E**–**H**) (*n* = 5–6 rats per group). (**A**) CeA *Pde4a* male Wistar*, t*_10_ = 2.28, *P* = 0.029. (**B**) CeA *Pde4a* female Wistar, *t*_10_ = 1.39, *P* = 0.09. (**C**) CeA *Pde4b* male Wistar, *t*_10_= 3.68, *P* = 0.004. (**D**) CeA *Pde4b* female Wistar, *t*_10_ = 2.53, *P* = 0.02. (**E**) CeA *Pde4a* male msP, *t*_10_ = 2.20*, P* = 0.02*.* (**F**) CeA *Pde4a* female msP, *t*_10_ = 1.23, *P* = 0.12. (**G**) CeA *Pde4b* male msP, *t*_10_*=* 2.73, *P* = 0.01*.* (**H**) CeA *Pde4b* female msP, *t*_10_ = 2.51, *P* = 0.01. All results are shown as mean ± SEM as well as individual values and analyzed as unpaired *t* tests. Significant difference relative to naive controls is denoted by *P < 0.05 and **P < 0.01.

Both CeA and NAc gene expression of *Pde4* subtypes *a* and *b* were assessed in subsets of naive and alcohol-exposed Wistar and msP rats. In the CeA, chronic alcohol exposure significantly increased *Pde4a* transcript levels in male Wistar and msP rats (Figure 7, A and E), but no differences were observed in females of either strain (Figure 7, B and F). Chronic alcohol drinking also significantly increased CeA *Pde4b* transcript levels in both male and female Wistar and msP rats (Figure 7, C, D, G, and H).

The same group of rats and genes were analyzed in the NAc. For *Pde4a*, chronic alcohol drinking significantly increased NAc *Pde4a* transcript levels in msP males (Supplemental Figure 3E) but not Wistar males (Supplemental Figure 3A). In contrast, alcohol decreased NAc *Pde4a* in females across both strains of rats (Supplemental Figure 3, B and F). Lastly, alcohol increased NAc *Pde4b* in Wistar male rats (Supplemental Figure 3C) but not the other strain or sex groups (Supplemental Figure 3 D, G, and H).

## Discussion

The present study investigated the effect of the PDE4 inhibitor apremilast on co-occurring alcohol drinking and mechanical allodynia in genetically selected msP rats and their Wistar counterparts. Based on prior work (12, 21), we hypothesized that PDE4 inhibition with apremilast would decrease alcohol drinking and alcohol-induced mechanical allodynia in rats. Apremilast decreased alcohol drinking in both strains and sexes, consistent with previous work (42). Apremilast also decreased mechanical allodynia during alcohol exposure, as well as during early (24 hours) and protracted (4 weeks) abstinence across strains and sex (but not in male Wistars). Our electrophysiological data show significant strain- and sex-dependent changes in baseline GABAergic transmission. Acute apremilast application increased presynaptic CeA GABA (sIPSC frequency) release, and GABA_A_ receptor function (i.e., sIPSC rise and decay) in male and female Wistar rats, respectively — an effect that was absent in msP groups. CeA *Pde4a* and *Pde4b* transcript levels were increased by alcohol across groups of sex and strain, similar to prior work in the striatum of animal models of binge-like drinking (12, 13). Collectively, these findings support the role of PDE4 in alleviating co-occurring AUD and increased pain sensitivity in both sexes.

Males and females of both strains displayed decreased alcohol drinking after administration with apremilast. This finding is in line with recent published reports testing PDE4 inhibitors across multiple animal models of AUD and selectively bred mouse strains for high alcohol intake (13, 21, 42, 43). Our results show that rats (both sexes, 2 strains) display similar reduction profiles in drinking behavior with apremilast. Other studies have examined sex differences with different classes of PDE4 inhibitors. For example, the PDE4 inhibitor rolipram decreased alcohol drinking in male and female High Drinking in the Dark (HDID, lines 1 and 2) mice and the genetically Heterogeneous Stock/Northport (HS/Npt) mice (44). A nonselective PDE inhibitor, ibudilast, decreased alcohol relapse behavior in rodents and in both male and female patients (45, 46). Specific subtypes such as PDE4A or -B have the ability to promote different roles in drinking between sexes. Studies with selective PDE4B inhibitors report mixed results, where Chavez et al. showed reductions in drinking in 2 sub-strains of mice and Blednov et al. found no effect on alcohol intake in mice but observed decreased alcohol-related ataxia (47, 48). The Blednov et al. report showed that a PDE4D inhibitor subtype decreased drinking in both male and female mice (47). Future studies are needed to characterize the role of specific PDE4 subtypes across sex and strain and their underlying mechanisms that reduce drinking.

Our work expands previous observations made in msP rats, a genetic animal model of excessive alcohol drinking that cosegregates with hyperanxiety and depressive-like conditions (49, 50). To our knowledge, there are no reports examining the effect of apremilast on alcohol-associated mechanical allodynia. However, apremilast has been shown to decrease autoimmune disease–related pain states (51). In our study, apremilast reduced mechanical allodynia across multiple phases of alcohol drinking, including early and protracted abstinence in both sexes and strains (but no effect in Wistar males). Differences in the efficacy of apremilast to reduce allodynia between male and female Wistars might depend on variation in their propensity to develop allodynia. We recently observed a higher tendency of female Wistars (compared with male) to develop allodynia in the 2-BC model, suggesting differences in the magnitude of pain and sensitivity for reducing pain with apremilast (24). We also note that msP and Wistar rats spontaneously drink different amounts of alcohol (msP taking more), which may influence the effects of apremilast on mechanical allodynia associated with alcohol exposure. While this is the first report of the effects of apremilast on co-occurring alcohol drinking and mechanical allodynia, other PDE4 inhibitors have been tested for non-alcohol-related pain behavior. Rolipram decreases neuropathic pain by reversing mechanical hypersensitivity in a mouse model of peripheral nerve injury (52). Selective knockdown of *Pde4b* via intrathecal injections of siRNA blocks pain hypersensitivity in a rat model of L5 spinal nerve ligation (53). Our research group recently reported changes in the dorsal root ganglion (DRG) endocannabinoid system in msP rats that also experienced persistent mechanical allodynia into late abstinence (24). In addition, Megat et al. (52) found that rolipram acts on nonneuronal glial cells of the DRG and exerts antiallodynic action and reduced expression of proinflammatory target TNF-α, suggesting that modulation of PDE4 for pain sensitivity occurs at the DRG level (52).

When we evaluated CeA *Pde4a* and *Pde4b* transcript levels, we found a generalized increase in their expression following alcohol, as similarly found in the striatum of animal models of binge-like drinking (12, 13). Additionally, we observed that apremilast increases spontaneous CeA GABA transmission in Wistar rats but not in msP rats. The effects of apremilast have been reported in other interconnected limbic structures, including the NAc. For instance, apremilast administration activates both excitatory and inhibitory synaptic inputs to the NAc MSN, with increased excitability of D1- but not D2-expressing NAc MSNs (12). Site-specific intra-NAc administration of apremilast is sufficient to decrease binge-like alcohol drinking in mice selectively bred for alcohol intoxication (12), pointing to a possible locus for NAc PDE4 in modulating the behavioral effects of alcohol drinking. It is also recognized that the role of PDE4 may involve other structures including the periaqueductal gray (PAG) due to connections with CeA to influence drinking and hyperalgesia (54, 55). CeA-PAG projections mediate thermal hyperalgesia in alcohol-dependent rats, and alcohol dependence reduced inhibition of PAG neurons evoked from CeA inputs (55). Future studies are needed to functionally validate involvement of CeA PDE4, including other structures in co-occurring drinking and alcohol-related mechanical allodynia.

We speculate that the effects of apremilast on the GABAergic signaling in CeA may occur through cAMP protein kinase A–dependent (PKA-dependent) mechanisms. β3-S408/409A male and female mice with a genetic modification that prevents PKA phosphorylation at GABA_A_ β3 subunits displayed no effective reduction in alcohol intake following apremilast administration versus WT mice as well as ataxia and loss-of-righting reflex (21). Similarly, the PKA inhibitor H-89 blocks the ability of apremilast to restore alcohol-induced ataxia (43). These data suggest that increased CeA GABA transmission by acute apremilast may involve β3 sites, presumably lost in msP rats. A recent report found that PDE4 dynamically changes across acute versus chronic alcohol exposure, where acute alcohol increases radioligand binding of PDE4B and where chronic alcohol decreases this binding (56). Our study observed increased CeA *Pde4b* transcript levels after chronic alcohol exposure, which may suggest a compensatory mechanism to facilitate increased cAMP/PKA signaling. Our model may contain differences in brain efflux transporter of PDE4s, splice gene variants of CeA PDE4A or PDE4B, or other intracellular signaling cascades that may not overlap with the decreased radioligand binding density of PDE4B as reported (56).

We recognize the potential sedation or nonspecific effects of apremilast may have influenced our results. We have previously explored this possibility in our related original work (12). Grigsby et al. (12) found that systemic apremilast decreases water and saccharin intake in HDID-1 and HDID-2 mice. This same report found that the site-specific injection of apremilast in the NAc did not alter water or saccharin intake levels (12). In the human study, apremilast did not produce adverse side effects, resulting in tolerability of this drug with no discontinuation in patients with AUD (12). In the current report, we did not observe changes in water intake at a time point when both drinking and mechanical threshold were tested with apremilast, supporting that animals were not sedated or displaying altered motivation for water across our groups/strains. Apremilast has been shown to have less severe PDE4 nonspecific affects compared with earlier PDE4 inhibitors (57). Another study also showed prolonged loss of righting reflex and recovery from alcohol in mice after apremilast (58). We note that the role of CeA in sedative or other nonspecific effects needs to be explored, given mixed results in systemic versus intra-NAc, strain differences, and species inconsistencies (rats versus mice).

Taken together, our findings show that apremilast decreases co-occurring drinking and mechanical allodynia across strain and sex. Acute apremilast also increases GABAergic transmission in the CeA of Wistars but not msP rats. Originally prescribed for psoriasis (commercialized as Otezla), the repurposing potential of apremilast has received significant interest as emerging work has proven its efficacy in reducing alcohol drinking across multiple animal models (12, 21, 42, 47). Promising human clinical studies conducted by several laboratories revealed that daily apremilast (90 mg) reduces the number of drinks and proportion of heavy drinking days relative to placebo controls in 51 non-treatment-seeking individuals with AUD in both men and women (12). Future clinical studies with apremilast for AUD could benefit from inclusion of measures of chronic pain and pain sensitivity.

## Methods

### Sex as a biological variable.

The present study included adult male (–450 g) and female (–250 g) Wistar (*n* = 25 male; *n* = 25 female) and genetically selected msP (*n* = 28 male, *n* = 21 female) rats. The rationale to include both sexes was to understand apremilast’s responsivity between male and female across our testing regimen. The msP rat colony was previously obtained from the School of Pharmacy at the University of Camerino; thus, the msPs and Wistars used in the present study were bred at Scripps Research. All rats were pair housed in a temperature- and humidity-controlled housing room that was maintained on a 12-hour reverse light-dark cycle (lights on 8 p.m.) with access to food and water ad libitum.

### Drugs.

For behavioral experiments, apremilast (Toronto Research Chemicals) was dissolved in 15% Tween 80 and 85% sterile saline and administered i.p. The doses of apremilast administered were 10 or 20 mg/kg chosen based on prior reports of effectively reducing drinking behavior in mice (12, 42, 58). Alcohol for 2-BC drinking sessions was prepared as 10% ethanol (v/v, 200 proof ethanol in drinking water). For electrophysiology experiments, apremilast was dissolved in 100% DMSO to first achieve 10 mM stock and then diluted fresh with artificial cerebrospinal fluid (aCSF) on recording days to yield final concentrations of 1 μM apremilast and < 0.03% DMSO in the bath.

### 2-BC alcohol drinking.

The 2-BC procedure (free choice between water and 10% v/v ethanol) was used to measure voluntary alcohol drinking and preference (59). Animals were first given free access to water and alcohol (10% v/v) 24 hours/day, for 15 consecutive days to establish a stable drinking baseline and preference for alcohol (80%–90% preference alcohol versus water in msP rats). Once this baseline was achieved, access to alcohol was decreased to 2 hours/day. Alcohol access started 2 hours into the animals’ dark, active phase for 4 consecutive days until a stable 2-hour–drinking baseline was reached as described previously (49). On the fifth day, rats were injected with either vehicle or apremilast (10 or 20 mg/kg), 1 hour before the beginning of the 2-BC session (2 hours). Apremilast was administered using a counter-balanced within-subject, Latin square design. Injections were administered every 7 days, and injection days were separated by at least three 2-BC drinking sessions. For all drinking sessions, fluids were offered through graduated drinking tubes equipped with metal sippers, and intake was measured by the difference in pre- and postsession drinking tube weight. The location of water and alcohol drinking tubes were switched daily to avoid the development of side preference. Alcohol intake was calculated as gram of alcohol consumed per kilogram of body weight, and preference was calculated by dividing the 10% alcohol intake by the total fluid (alcohol + water) intake and multiplying by 100. Blood alcohol levels (BALs) were not recorded; however, the expression of high alcohol intake in the msP strain is similar to our original work characterizing the rodent line (36, 49, 60). The drinking levels produced by Wistars is approximately 50% preference, and msPs are approximately 80%–90% preference, with msPs resulting in approximately 70–80 mg/dL BALs and peaking over 100 mg/dL (60).

### von Frey test for mechanical sensitivity.

Mechanical sensitivity was measured using von Frey filaments (North Coast Medical) by the same blinded experimenter throughout the study, as previously reported (24). The animals were tested under red lights, during their dark (active) phase. Briefly, rats were first habituated to the testing apparatus by placing them on a metal mesh stand for 15 minutes. von Frey filaments (4, 6, 8, 10, 15, 26, 60, and 100 g) were then manually applied to the plantar surface of 1 hind paw with increasing force until a positive withdrawal response was elicited. A positive withdrawal response was recorded if the paw was lifted at least 3 times in 5 replicate measurements of the same force. If a positive withdrawal response was not recorded, the next filament with a greater force was tested. The lowest von Frey filament force that elicited a positive withdrawal response was recorded as the mechanical withdrawal threshold. A baseline measurement of each animal was taken immediately following the 2-BC session (2 hours) the day before treatment with apremilast. On injection days, after the 2-hour 2-BC session (i.e., 3 hours after i.p. drug injection), vehicle or apremilast-treated (10 and 20 mg/kg) Wistar and msP rats were tested again for mechanical sensitivity. The vehicle and 20 mg/kg injected rats were also tested 24 hours after injection (early abstinence). The focus to only test the 20 mg/kg dose of apremilast in early abstinence was based on our finding that the 10 mg/kg dose of apremilast did not produce any significant effects in mechanical threshold on injection day. We previously observed that male and female msP rats developed persistent mechanical allodynia during protracted abstinence from alcohol, while Wistar rats did not (24). This finding was confirmed herein (Supplemental Figure 2). For this reason, apremilast (20 mg/kg) was additionally tested in msP male and female rats 4 weeks after alcohol removal in the same manner used previously (i.e., baseline recording the day before apremilast injection, measurement taken 3 and 24 hours after apremilast injection). Herein, the term mechanical allodynia is used to describe withdrawal thresholds that are lower than that of baseline levels.

### CeA electrophysiology.

A random subset of 3–4 rats per group were used for electrophysiology as previously described (40, 61, 62). Rats were anesthetized with 3%–5% isoflurane and euthanized right at the end of their last 2-hour 2-BC session. Brains were quickly isolated and placed in ice-cold oxygenated (95% O_2_ and 5% CO_2_) high-sucrose cutting solution (in mM: sucrose, 206; KCl, 2.5; CaCl_2_, 0.5; MgCl_2_, 7; NaH_2_PO_4_, 1.2; NaHCO_3_, 26; glucose, 5; HEPES, 5). Coronal slices (300 μm) containing the CeA were cut on a Leica 1200S vibratome (Leica Microsystems) and then incubated in artificial cerebrospinal fluid (in mM: NaCl, 130; KCl, 3.5; NaH_2_PO_4_, 1.25; MgSO_4_ × 7H_2_O, 1.5; CaCl_2_, 2.0; NaHCO_3_, 24; glucose, 10) at 37ºC for 30 minutes and then at room temperature for a minimum of 30 minutes. We recorded from a total of 50 neurons in the medial subdivision of the CeA that were visualized with infrared differential interference contrast optics and CCD cameras (QImaging). Recordings were performed in gap-free acquisition mode with a sampling rate per signal of 20 kHz and low-pass filtered at 10 kHz, using a Multiclamp 700B amplifiers, Digidata 1440A digitizer, and pClamp 10 software (Molecular Devices). We filled borosilicate glass patch pipettes (3–6 MΏ; Warner Instruments) with internal solution (in mM: 145.0 KCl; 5.0 EGTA; 0.5 MgCl_2_; 10.0 HEPES; 2.0 Mg-ATP; 0.2 Na-GTP, adjusted with 1M KOH [pH 7.2–7.4]). sIPSCs were pharmacologically isolated with 6,7-dinitroquinoxaline-2,3-dione (DNQX, 20 μM), DL-2-amino-5-phosphonovalerate (DL AP-5, 30 μM), and CGP55845A (1 μM). Neurons were clamped at –60 mV, and experiments with a series resistance of the recording pipettes > 25 MΏ or a > 20% change in series resistance, as monitored with frequent 10 mV pulses, were excluded. All recordings were performed at room temperature. All sIPSC frequency, amplitude, and kinetics (rise and decay times) data were analyzed using Mini Analysis (Synaptosoft Inc.) and visually confirmed. Only sIPSCs > 5 pA were accepted for analysis, and their characteristics were binned based on 3–5 minutes of recording. Apremilast (1 μM) was applied onto each CeA slice for 12–16 minutes, and the maximum value recorded during minutes 12–15 of drug application was used for analysis (12). The concentration of apremilast (1 μM) was based on previous electrophysiology experiments in mice (12).

### CeA gene expression.

This study was performed in a subset of msP and Wistar rats (*n* = 5–7 per group) that were kept under 2-BC alcohol drinking for several weeks after receiving vehicle or apremilast injections. Male and female msP and Wistar alcohol-exposed rats were anesthetized with isoflurane and rapidly decapitated at the end of the 2-hour 2-BC session. Brains were harvested, flash frozen in dry ice–cooled isopentane, and stored at −80°C until analysis. The CeA and NAc were dissected from coronal cryostat sections (400 μm) using a stainless-steel punch. RNA was isolated from the CeA and NAc using Trizol (Invitrogen; catalog 15596026) and an RNA extraction kit (Zymo Research; catalog NC9972645). cDNA synthesis was conducted using SuperScript IV exDNAse kit (Invitrogen; catalog 11766050). cDNA was amplified using SYBR green PowerTrack master mix (Applied Biosystems; catalog A46109) and quantified by quantitative PCR (qPCR) on the QuantStudio 5 system. Each sample was loaded in duplicate. Cycling conditions were as follows: 95°C denaturation temperature for 15 seconds, 60.3°C annealing temperature for 15 seconds, and 72°C extension temperature for 15 seconds. The BestKeeper software (Version 1, 2003, gene-quantification) was used to determine the expression stability and the geometric mean of 2 different housekeeping genes (*Actb* and *Ywhaz*) (63, 64). The relative expression of genes of interest was measured by the 2^−ΔΔCT^ method (65), where ΔCT was calculated by subtracting the CT value of the geometric mean of the housekeeping genes from the CT value of the gene of interest. *Pde4a* and *Pde4b* fold changes are expressed relative to naive controls. Primers were obtained from Integrated DNA Technologies. See Table 1 for all forward and reverse primer sequences.

### Experimental approach.

The present study was conducted in 2 separate rat cohorts. A timeline of the approach was provided in Figure 1. Cohort 1 tested the effects of apremilast (0, 10, 20 mg/kg) on 2-BC alcohol drinking and mechanical allodynia using von Frey across strain and sex (Figure 2 and Figure 3; tested on weeks 3–5). Cohort 2 tested the effects of apremilast (0 or 20 mg/kg) on mechanical allodynia only into protracted abstinence (Figure 4; tested on week 9). Cohort 2 did not receive alcohol again after their last week of drinking (week 5). Subsets of rats from cohort 1 were examined for gene expression changes in *Pde4* subtypes *a* or *b* in the CeA and NAc (tissue collected on week 7) as well as electrophysiological recording in the CeA (recording on week 7 and 8).

### Statistics.

Sex and strain were analyzed independently in the behavioral data using 1- or 2-way ANOVA models. 2-BC drinking data were analyzed using 1-way ANOVA with drug (apremilast: 0, 10, or 20 mg/kg) as a factor. For the von Frey data, test for assumptions of normality were applied, and in cases where violations of normality occurred, a repeated measures (RM) 1-way ANOVA or mixed-effects analysis using a Greenhouse-Geisser correction factor to adjust degrees of freedom resulting in more accurate *F*-ratios (immediately after alcohol) or nonparametric Wilcoxon signed-rank test (early abstinence) was used. Time course von Frey data were analyzed using a RM 2-way ANOVA with drug as a factor and time as a within-subjects factor. For electrophysiology data, baseline (predrug) differences in sIPSC parameters, including sIPSC frequencies, amplitudes, rise time, and decay time were analyzed as an independent samples 2-tailed *t* test to compare strain. If the data had equal variance and normal distribution according to the Kolmogorov-Smirnov test, the acute effect of apremilast on sIPSC parameters was expressed relative to baseline (100%), and changes were evaluated by 1-sample *t* test. In cases where ANOVAs were significant, follow-up post hoc analyses adjusted for multiple comparisons using Dunnett’s correction or Tukey’s HSD test were used. Gene expression data for *Pde4a* and *-4b* were analyzed as 1-tailed *t* tests in alcohol-exposed rats versus naive. The rationale to use a 1-tailed *t* test was based on previous work showing increases in these genes after binge-like alcohol intake and lower sample size in these data sets (12). We hypothesized similar direction in genetically selected msP rats across our drinking regimen. To understand interaction effects of sex, strain, and drug or alcohol, we have included a separate analysis with a full factorial ANOVA model in Supplemental Table 1. Significance α level was set at *P* < 0.05.

All experimenters were blind to the subjects’ treatment conditions during testing and sample preparations. All data were analyzed and graphed using GraphPad Prism 10 software. Five rats were excluded from von Frey analysis due to aberrant locomotor behaviors.

### Study approval.

All experimental procedures were approved by Scripps Research IACUC (protocol no. 09-0006 and 24-0008) and followed the *Guide for the Care and Use of Laboratory Animals* (National Academies Press, 2011).

### Data availability.

All data are available upon request. Values for all data points in graphs are reported in the Supporting Data Values file.

## Author contributions

MR, VV, VB, and BC designed research studies. VV, VB, MB, RB, CMSO, DGC, and RV conducted experiments, acquired data, and analyzed data. ARO provided reagents. MR, BC, CMSO, RC, ARO, VV, VB, and AJR all contributed to writing the manuscript, graphing, and intellectual input of data.

## Supplementary Material

Supplemental data

Supporting data values

## Figures and Tables

**Table 1:**
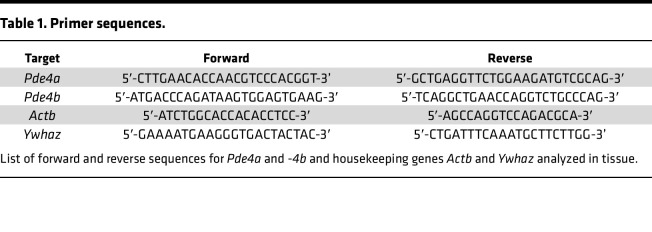
Primer sequences.

## Version 1 04/22/2025

Electronic publication
