# Supplementary material for: Apremilast reduces co-occurring alcohol drinking and mechanical allodynia and regulates central amygdala GABAergic transmission
Source: JCI Insight. 2025 Apr 22;10(8):e189732. doi: 10.1172/jci.insight.189732 (PMC12016922; doi:10.1172/jci.insight.189732)
Supplement: Supplemental data [file jciinsight-10-189732-s171.pdf]

Supplementary Materials & Results

Title: Apremilast reduces co-occurring alcohol drinking and mechanical allodynia and regulates central amygdala GABAergic transmission

Authors: Valentina Vozella[1‡], Vittoria Borgonetti[1‡], Bryan Cruz[1‡], Celsey M. St. Onge[1], Ryan Bullard[1], Roman Vlkolinsky[1], Diego Gomez-Ceballos[1], Angela R. Ozburn[2], Amanda J. Roberts[3], Roberto Ciccocioppo[4], Michal Bajo[1], & Marisa Roberto[1]

‡ Equal Contribution

Affiliation: [1]Department of Translational Medicine, Scripps Research, La Jolla, California, USA; [3]Animal Models Core Facility, Scripps Research, 10550 North Torrey Pines Road, La Jolla, CA 92037, USA; [2]Department of Behavioral Neuroscience at Oregon Health & Science University and VA Portland Health Care System, Portland, OR, 97239, USA; [4]Pharmacology Unit, School of Pharmacy, University of Camerino, Camerino, Italy.

Correspondence: Marisa Roberto, Ph.D., Department of Translational Medicine, Scripps Research; 10550 N. Torrey Pines Road, La Jolla, CA 92037, Email: mroberto@scripps.edu

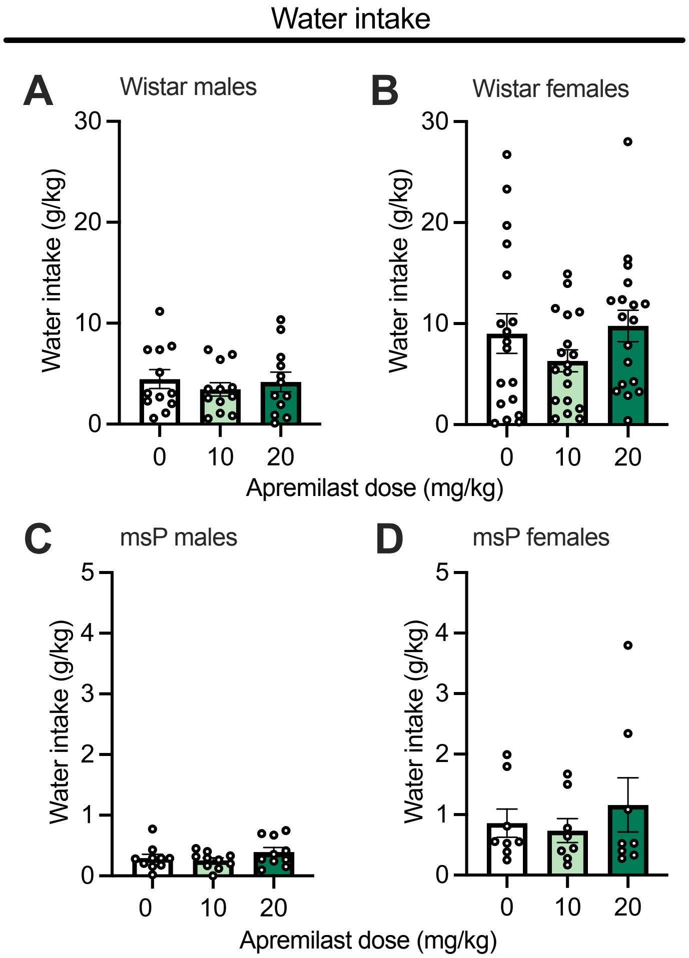

Figure S1. Effect of apremilast (10 or 20 mg/kg) on water intake in the 2-BC drinking procedure (10% *v/v* alcohol) in Wistar (**A,B**: top row) and msP (**C,D**: bottom row) rats. (**A**) Wistar male water intake, $F_{2,33}$ = 0.36, P = 0.69, (**B**) Wistar female water intake, $F_{2,51}$ = 1.31, P = 0.27, (**C**) msP male water intake, $F_{2,27}$ = 1.33, P = 0.27, and (**D**) msP female water intake, $F_{2,21}$ = 0.48, P = 0.62. Results are expressed as mean ± SEM and analyzed as one-way ANOVA.

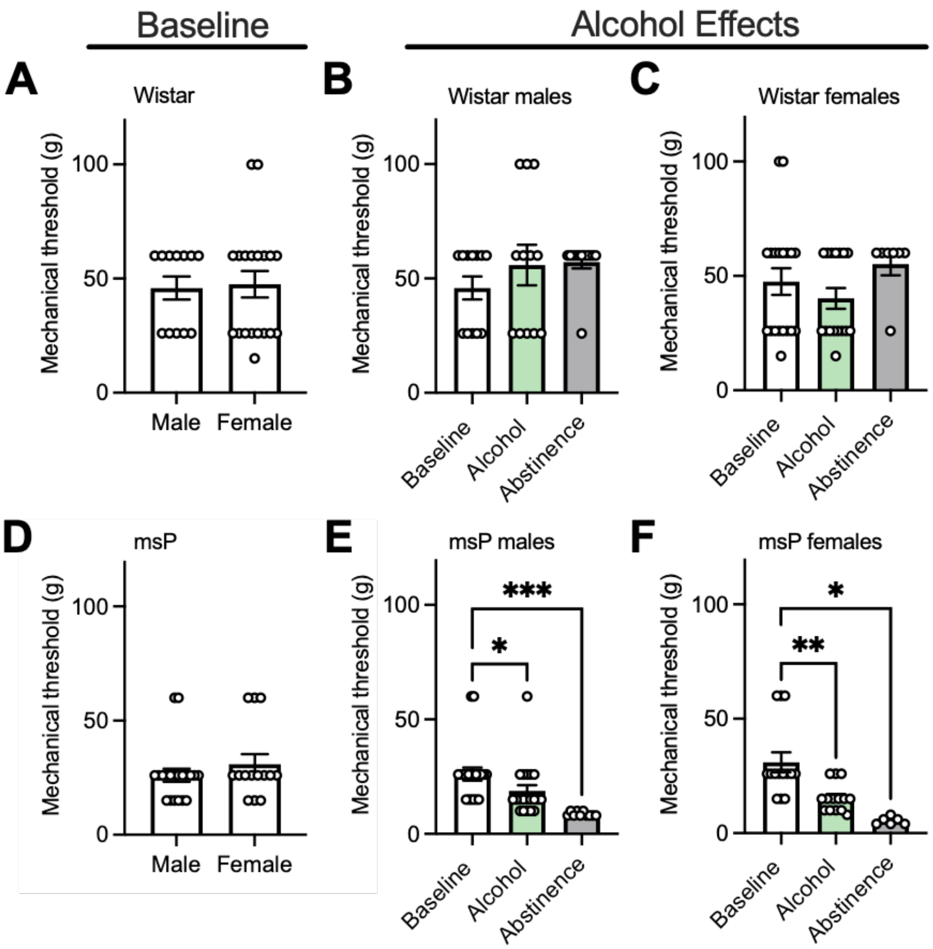

Figure S2. Baseline and alcohol effects on mechanical allodynia in Wistar and msP rats before starting 2BC (Baseline), immediately after 2BC (Alcohol; 2 weeks of 24h/2BC) and after long abstinence (Abstinence; 4 weeks from alcohol-removal). (**A**) Baseline mechanical threshold between Wistar male and female, U = 112, P > 0.999. (**B**) Alcohol effects on mechanical threshold in Wistar males, $F_{1.526,16.78} = 0.91$, P = 0.393. (**C**) Alcohol effects on mechanical threshold in Wistar females, $F_{1.656,17.39} = 1.08$, P = 0.347. (**D**) Baseline mechanical threshold between Wistar male and female, U = 118, P = 0.405. (**E**) Alcohol effects on mechanical threshold in msP males, $F_{1.970,27.58} = 14.11$, P < 0.0001. (**F**) Alcohol effects on mechanical threshold in msP females, $F_{1.179,10.61} = 14.44$, P = 0.0024. Results are expressed as mean ± SEM and analyzed as a repeated measures one-way ANOVA or mixed-effects analysis as appropriate with Geisser-Greenhouse correction followed by Dunnet's multiple-comparison *post hoc* test. Significant difference relative to vehicle controls is denoted by *$p < 0.05$, **$p < 0.01$ and *** $p < 0.001$.

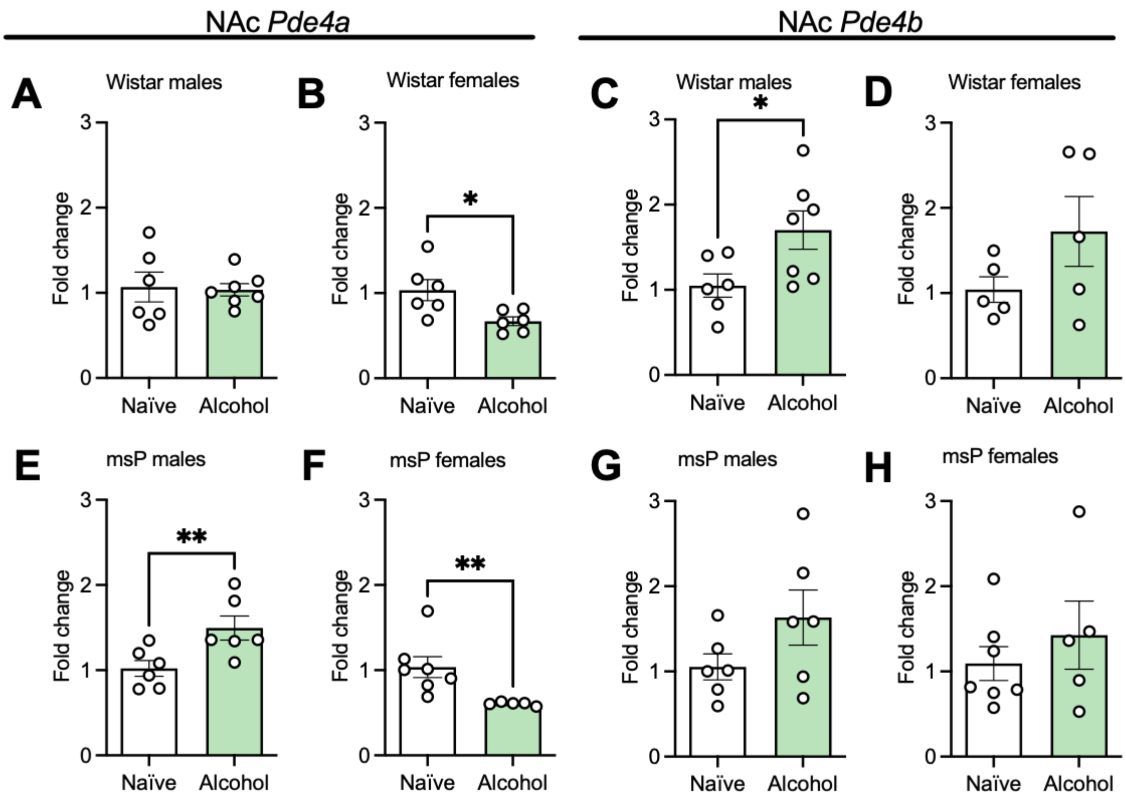

Figure S3. NAc *Pde4* subtype *a* and *b* transcript levels are altered by chronic alcohol exposure. Effect of chronic alcohol exposure (7 weeks) in NAc *Pde4* subtypes *a* and *b* in Wistar and msP rats. NAc *Pde4a* (left panels) and *Pde4b* (right panels) in male and female Wistar (**A-D:** top row) and msP rats (**E-H:** bottom row). (**A**) NAc *Pde4a* male Wistar, t_{11} = 0.17, P = 0.431, (**B**) NAc *Pde4a* female Wistar, t_{10} = 2.73, P = 0.011, (**C**) NAc *Pde4b* male Wistar, t_{11} = 2.37, P = 0.018, (**D**) NAc *Pde4b* female Wistar, t_{8} = 1.56, P = 0.078, (**E**) NAc *Pde4a* male msP t_{10} = 2.80, P = 0.009, (**F**) NAc *Pde4a* female msP, t_{10} = 2.92, P = 0.008, (**G**) NAc *Pde4b* male msP, t_{10} = 1.62, P = 0.068, and (**H**) NAc *Pde4b* female msP, t_{10} = 0.81, P = 0.217. All results are shown as mean ± SEM as well as individual values and analyzed as unpaired t-tests. Significant difference relative to naïve controls is denoted by * defined as $p < 0.05$ and ** defined as $p < 0.01$.

Table S1. Full Factorial ANOVA Models

Figure	Dependent Variable	ANOVA Model	Effect Size
2	Alcohol Intake	3-Way	*Drug: $F_{2,131}$ = 3.57, p < 0.001<br>*Sex: $F_{1,131}$ = 3.99, p = 0.048<br>*Strain: $F_{1,131}$ = 348.18, p < 0.001<br>Drug × Sex × Strain: $F_{2,131}$ = 0.85, p = 0.428
2	Alcohol Preference	3-Way	*Drug: $F_{2,131}$ = 4.23, p = 0.017<br>*Sex: $F_{1,131}$ = 7.24, p = 0.008<br>*Strain: $F_{1,131}$ = 218.22, p < 0.001<br>Drug × Sex × Strain: $F_{2,131}$ = 0.56, p = 0.568
3	Mechanical Threshold: Immediately After Alcohol	3-Way	*Drug: $F_{2,131}$ = 5.11, p = 0.007<br>Sex: $F_{1,131}$ = 0.20, p = 0.650<br>* Strain: $F_{1,131}$ = 18.37, p < 0.001<br>Drug × Sex × Strain: $F_{2,131}$ = 2.08, p = 0.128
3	Mechanical Threshold: Early abstinence	3-Way	*Drug: $F_{1,85}$ = 11.31, p = 0.001<br>Sex: $F_{1,85}$ = 0.20, p = 0.651<br>*Strain: $F_{1,85}$ = 16.28, p < 0.001<br>Drug × Sex × Strain: $F_{1,85}$ = 0.70, p = 0.402
4	Mechanical Threshold: Protracted abstinence	3-Way	*Drug: $F_{1,28}$ = 7.20, p = 0.012<br>Sex: $F_{1,85}$ = 0.588, p = 0.450<br>*Test Timepoint: $F_{2,56}$ = 6.40, p = 0.003<br>Test Timepoint × Drug × Sex: $F_{2,56}$ = 1.74, p = 0.184
5 & 6	Baseline: sIPSC Frequency	2-Way	Sex: $F_{1,46}$ = 2.04, p = 0.160<br>Strain: $F_{1,46}$ = 0.74, p = 0.391<br>*Sex × Strain: $F_{1,46}$ = 10.33, p = 0.002
5 & 6	Baseline: sIPSC Amplitude	2-Way	Sex: $F_{1,46}$ = 3.00, p = 0.090<br>Strain: $F_{1,46}$ = 0.00, p = 0.987<br>Sex × Strain: $F_{1,46}$ = 0.01, p = 0.918
5 & 6	Baseline: sIPSC Rise	2-Way	*Sex: $F_{1,46}$ = 8.30, p =0.006<br>*Strain: $F_{1,46}$ = 5.33, p = 0.025<br>Sex × Strain: $F_{1,46}$ = 1.12, p = 0.294
5 & 6	Baseline: sIPSC Decay	2-Way	Sex: $F_{1,46}$ = 2.56, p = 0.116<br>Strain: $F_{1,46}$ = 1.95, p = 0.169<br>Sex × Strain: $F_{1,46}$ = 0.006, p = 0.938
5 & 6	Apremilast: sIPSC Frequency	2-Way	Sex: $F_{1,40}$ = 0.50, p = 0.483<br>Strain: $F_{1,40}$ = 0.64, p = 0.426<br>Sex × Strain: $F_{1,40}$ = 1.82, p = 0.184
5 & 6	Apremilast: sIPSC Amplitude	2-Way	Sex: $F_{1,40}$ = 0.52, p = 0.474<br>Strain: $F_{1,40}$ = 1.69, p = 0.200<br>Sex × Strain: $F_{1,40}$ = 0.491, p = 0.487

5 & 6	Apremilast: sIPSC Rise	2-Way	Sex: $F_{1,40}$ = 0.14, p = 0.703<br>Strain: $F_{1,40}$ = 0.84, p = 0.363<br>Sex × Strain: $F_{1, 40}$ = 3.04, p = 0.089
5 & 6	Apremilast: sIPSC Decay	2-Way	Sex: $F_{1,40}$ = 0.02, p = 0.889<br>Strain: $F_{1,40}$ = 1.20, p = 0.278<br>Sex × Strain: $F_{1,40}$ = 0.28, p = 0.595
7	CeA *Pde4a:* Fold Change	3-Way	Sex: $F_{1,40}$ = 0.86, p =0.358<br>Strain: $F_{1,40}$ = 1.29, p = 0.262<br>*Alcohol: $F_{1,40}$ = 17.34, p < 0.001<br>Sex × Strain × Alcohol: $F_{1,40}$ = 0.50, p = 0.483
7	CeA *Pde4b:* Fold Change	3-Way	Sex: $F_{1,40}$ = 1.89, p = 0.177<br>Strain: $F_{1,40}$ = 0.38, p = 0.539<br>*Alcohol: $F_{1,40}$ = 28.91, p < 0.001<br>Sex × Strain × Alcohol: $F_{1,40}$ = 0.06, p = 0.794
S1	Water Intake	3-Way	Drug: $F_{2,132}$ = 17.57, p = 0.415<br>*Sex: $F_{1,132}$ = 10.14, p = 0.002<br>*Strain: $F_{1,132}$ = 51.88, p < 0.001<br>Drug × Sex × Strain: $F_{2,132}$ = 0.21, p = 0.810
S2	Baseline Difference: Mechanical Threshold	2-Way	Sex: $F_{1,61}$ = 0.47, p = 0.495<br>*Strain: $F_{1,61}$ = 14.60, p < 0.001<br>Sex × Strain: $F_{1,61}$ = 0.10, p =0.743
S2	Alcohol Differences: Mechanical Threshold	2-Way	Sex: $F_{1,150}$ = 1.10, p = 0.294<br>*Strain: $F_{1,150}$ = 136.12, p < 0.001<br>Alcohol: $F_{2,150}$ = 1.97, p = 0.142<br>Sex × Strain × Alcohol: $F_{1,150}$ = 0.51, p = 0.598
S3	NAc *Pde4a:* Fold Change	3-Way	*Sex: $F_{1,41}$ = 15.91, p < 0.001<br>Strain: $F_{1,41}$ = 1.21, p = 0.276<br>Alcohol: $F_{1,42}$ = 1.22, p = 0.275<br>Sex × Strain × Alcohol: $F_{1,41}$ = 3.19, p = 0.081
S3	NAc *Pde4b:* Fold Change	3-Way	Sex: $F_{1,39}$ = 0.43, p = 0.837<br>Strain: $F_{1,39}$ = 0.17, p = 0.675<br>*Alcohol: $F_{1,39}$ = 9.34, p = 0.004<br>Sex × Strain × Alcohol: $F_{1,39}$ = 0.14, p = 0.707
